# How do healthcare unit managers promote nurses' perceived organizational support, and which working conditions enable them to do so? A mixed methods approach

**DOI:** 10.1111/sjop.12851

**Published:** 2022-06-30

**Authors:** Christian Gadolin, Pernilla Larsman, Maria Skyvell Nilsson, Anders Pousette, Marianne Törner

**Affiliations:** ^1^ Department of Health Sciences University West Trollhättan Sweden; ^2^ Department of Psychology University of Gothenburg Gothenburg Sweden; ^3^ School of Public Health and Community Medicine, Institute of Medicine, Sahlgrenska Academy University of Gothenburg Gothenburg Sweden

**Keywords:** Healthcare administration, healthcare leadership, human resource management, mixed methods, organizational climate, psychosocial working conditions

## Abstract

Healthcare unit managers are pivotal to promote nurses' Perceived Organizational Support and hence to ensure nurses' health and well‐being, as well as high‐quality care. Despite this fact, there is a dearth of studies addressing how healthcare unit managers act and organize their work to promote nurses' Perceived Organizational Support and which working conditions enable them to do so. Through a mixed methods approach, comprising qualitative interviews and quantitative surveys among healthcare unit managers and nurses, this paper underscores that healthcare unit managers' availability to their nursing staff was essential for their ability to promote nurses' Perceived Organizational Support, and that responsive support from the care unit managers' superior management, administration, and managerial colleagues constituted enabling working conditions. Superior manager support strongly promoted the care unit manager's own Perceived Organizational Support, which, in turn, was positively correlated with nurses' organizational climate of Perceived Organizational Support.

## INTRODUCTION

Efficient delivery of healthcare services is dependent on the health and well‐being of the healthcare professionals (Demerouti et al., 2001; Eklöf et al., 2014), but increasing work‐related ill‐health among healthcare staff is widely noted (Bridgeman et al., 2018; O'Keeffe et al., 2015). Increased perceived stress, burnout, and lower job satisfaction have been established among nurses (Aamir et al., 2016; Almada et al., 2004; Johnson et al., 2016; Weber, 2010; Whitehead et al., 2015). This threatens the ability of healthcare organizations to recruit and retain nurses, drives costs and negatively affects the quality of the care (Coomber and Barrinall, 2007; Cowin, 2002; Flinkman et al., 2010; Heinen et al., 2013; Sasso et al., 2019; Tourangeau and Cranley, 2006). This calls for knowledge about how healthcare organizations can be better managed to promote nurses' mental health and job satisfaction. Research has shown that employees' Perceived Organizational Support (POS) is negatively related to stress and burnout, and positively related to job satisfaction and an intention to stay in the organization, as well as to work commitment and performance (Kurtessis et al., 2017; Riggle et al., 2009). Therefore, promoting nurses' POS appears as a viable route in such a positive direction. Previous quantitative studies have identified general antecedents to POS (Ahmed et al., 2015; Kurtessis et al., 2017; Rhoades and Eisenberger, 2002; Riggle et al., 2009) that may guide organizational development. However, effective development of POS is also dependent on fine‐grained knowledge on contextual preconditions for this, specific for a sector, and distinct professional groups within the sector (cf. Ahmed et al., 2015; Paul and Phua, 2011; Rhoades and Eisenberger, 2002; Riggle et al., 2009). This calls for qualitative studies to contextualize the POS antecedents. In one of very few such studies, Gadolin et al. (2021) described organizational structures and structuration that supported nurses' POS. The study also highlighted the importance for nurses' POS, of the organization conveying individual recognition and professional acknowledgement, and offered contextualized descriptions of how this could be done convincingly. Others have shown that support from leaders, especially immediate superiors, is vital for nurses' job satisfaction (e.g., Cameron et al., 2004; Coomber and Barriball, 2007; Fang, 2001; Li and Lambert 2008). In concordance with this, Gadolin et al. (2021) identified the care unit (CU) manager as a central actor for providing organizational support to the nurses. However, research to clarify how CU managers act and organize their work to enable them to promote nurses' POS is lacking, as is knowledge about what organizational preconditions the CU managers themselves need in order to be able to act effectively in such a manner.

The aims of this study were to investigate (1) how CU managers act and organize their work to convey organizational support that promotes the development of nurses' POS; (2) the type of organizational support the CU managers need to provide such support, and the conditions that hinder such leadership; (3) if CU managers' own POS is related to nurses' organizational climate of POS; and (4) the demands and resources in CU managers' work situation that influence their own POS.

## THEORETICAL FRAMEWORK

*POS* is defined as employees' perceptions that the organization cares about their well‐being and appreciates their contribution to the organization (Eisenberger et al., 1986). POS theory is based on fundamental human socioemotional needs of social status, self‐esteem, and meaning. Its development is based on a personification of the organization. Actions taken by organizational agents, such as managers, are not interpreted solely as indications of the intent of the agent, but as the intent of the organization (Eisenberger et al., 1986; Rhoades and Eisenberger, 2002). Thus, managers' behavior, and not least that of the immediate supervisors, informs the employees about the extent to which the organization cares about their well‐being and appreciates their contributions.

*Social exchange theory* (Blau, 1964) stipulates that when a party in a social exchange receives a valuable resource from the other party, this will motivate the recipient to reciprocate. Working conditions that fulfill employees' fundamental needs of meaning and self‐worth, promote their well‐being, and allow them to perform their work well, constitute a valuable resource. Thus, an organization that provides its employees with such workings conditions will motivate them to contribute to the organizational goals and perform their job to the best of their ability. In concordance with theory on POS and social exchange, therefore, we suggest that CU managers who personally experience high POS will be more motivated and better able to structure and otherwise lead the work at the CU. Such effective leadership includes conveying a high level of care for the employees' well‐being and appreciation for their contribution. Thus, high CU manager POS would be expected to be associated with a high level of nurses' POS.

The *demand‐resource theory* (Bakker and Demerouti, 2007; Bakker et al., 2003) describes two general categories of the working situation: job demands and job resources. Job demands refer to “those physical, psychological, social, or organizational aspects of the job that require sustained physical and/or psychological (cognitive and emotional) effort or skills and are therefore associated with certain physiological and/or psychological costs” (Bakker and Demerouti, 2007, p. 312). Job resources refer to the corresponding organizational aspects of the job that are stimulating and reduce physiological and psychological costs (Bakker and Demerouti, 2007). The JD‐R model proposes two processes. One is driven by high demands and leads to job strain and health impairment. In the other, job resources lead to high work engagement and performance. Based on the JD‐R model, we propose that the CU managers' job demands and resources influence their POS, as well as their capability to convey organizational support to their employees.

## METHODS

The study approaches complex interactions between different organizational levels and parties, where some issues may be approached deductively and quantitatively, while others are little previously known and call for an inductive, interpretive, and contextualized approach. Therefore, we chose a mixed‐methods study design, which allows quantitative investigation in larger but sector‐specific samples of relations between factors that, based on theory and existing empirical research, may be hypothesized as consequential to the subject matter. Such studies need to be complemented by in‐depth, qualitative studies to better understand the social and individual mechanisms. The combination of quantitative and qualitative research may validate the results from each method and allows comparison of the results that may reveal contrasts that are important to understand the subject matter.

There are a great number of different typologies of mixed‐methods designs (see, e.g., Bishop, 2015) and no single agreed‐upon classification. However, by focusing on the central concepts of timing and emphasis, the present study can be described as a sequential integrated (Giddings and Grant, 2006) and explanatory sequential (Creswell and Plano Clark, 2011) design, with two distinct research phases. The qualitative and quantitative methods were considered to be of equal status.

*Phase 1* was directed to aims 1 and 2 and approached qualitatively, through in‐depth interviews with CU managers. *Phase 2* was directed toward aims 2, 3, and 4 through questionnaire surveys.

### Phase 1: Qualitative interviews with CU managers

#### Participants

Twenty CU managers from 11 medical specialties, in six different hospitals, across two regional Swedish public healthcare organizations, were interviewed (see Table 1). For selection of the informants, one of the researchers prepared a nine‐cell matrix with CU managers from both participating healthcare organizations based on the results of the questionnaires. One axis of the matrix represented CU managers self‐rated, individual POS (high, medium, or low). The other axis represented nurses' organizational climate of POS, aggregated to the respective CU (high, medium, and low). Each cell contained 1–6 CU managers. Informants were selected from each cell in order to obtain a sample with maximum purposeful variation (see Patton, 2002). Five managers did not wish to participate, meaning that one cell (medium manager POS/high nurses' POS climate) could not be represented. Between one and four CU managers in each of the remaining eight matrix cells were interviewed. The researchers conducting the interviews were blinded regarding which matrix cell each informant was derived from. Information regarding the study and its purpose was sent by email to the potential participants before the interview.

**TABLE 1:** Description of the care unit managers participating in the qualitative interviews

Number of informants (per geographical region)	N = 20 (Region X: 11, Region Y: 9)
Age, years; mean (SD)	52.0 (9.8)
Gender	F: 18, M: 2
Experience as a nurse, years; mean (SD)	19.0 (12.6)
Experience as a CU manager, years; mean (SD)	9.4 (9.4)
Manager of care unit, medical speciality (n)	Psychiatric acute care (1); cardiology (1); oncology (1); surgery (3); ambulance (3); geriatrics/neurology (1); psychiatric care (4); forensic psychiatry (2); internal medicine (2); acute surgery (1); urology (1)

#### Design and analysis

**Fig. 1:**
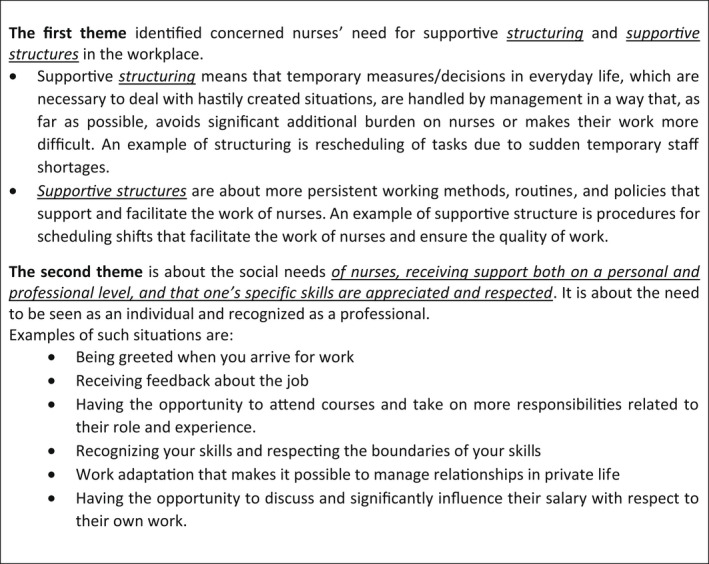
Summary of the results of the previously performed qualitative interviews with nurses.

All interviews were carried out remotely, either by phone or computer through the Zoom software. Despite the COVID‐19 pandemic, this allowed some personal interaction between interviewers and informants. The interviews (which lasted between 37 and 78 min) were guided by previous research (Gadolin et al., 2021), presenting the results of in‐depth, qualitative interviews with registered nurses in the two participating healthcare organizations. These results are presented briefly in Fig. 1. The CU manager interviews were recorded and transcribed verbatim.

The interviews were conducted by two of the authors (CG and MSN), both of whom have extensive experience of qualitative research. For the first 15 interviews, the critical incident technique (CIT) (Flanagan, 1954) was applied. This method limits the influence of the interviewer's subjectivity and preunderstandings (Druskat and Wheeler, 2003; Grill and Nielsen, 2019). It also allows the informants better access to context‐specific memories and personal experiences of situations relevant to the subject matter (Wheeler et al., 1997). The first 15 interviews were conducted in spring 2020. Initially, a summary of the thematic results from the nurse interviews (Fig. 1) was presented to the CU managers. The informant was then asked to choose one of the themes and describe a situation when he or she had been able to work supportively regarding the theme. The informants described what they had done and the contextual conditions that had enabled and supported this work. Only follow‐up questions were asked. When no more information on this situation could be retrieved, the informant was asked to select another theme and expand upon it in the same way. This was repeated until no more theme was selected. Subsequently, the procedure was repeated, where the CU manager was requested to select a theme and comprehensively describe situations and circumstances when he or she had been unable to work in a manner that was supportive to this theme. Here, too, the procedure was repeated until the informant presented no more situations.

A preliminary analysis of the 15 interviews indicated that the quality of the support from three different parts and levels of the organization were central to the CU manager's ability to contribute to nurses' POS: the superior managers, the administrative healthcare departments, and the peer CU managers. In the following five interviews, conducted in autumn 2020, we sought fuller and deeper descriptions regarding the CU managers' experience of support and lack of support from these parties, and how this had affected their ability to offer the support required by the nurses.

When all the interviews had been conducted, the complete text material was analyzed through qualitative content analysis to systematize the material into descriptive categories (Graneheim and Lundman, 2004). Initially, all text was read by three of the authors (CG, MSN and MT) to create an overall understanding of the content. Through this, and subsequent discussions within the research team, preliminary categories describing different organizational levels central to CU manager's ability to contribute to nurses' POS were identified. Subsequently, statements that related to the same central meaning were identified and sorted into these categories. The analysis was continuously discussed among the researchers through an iterative process until consensus on categorization, content, and interpretation was reached and consistency ensured.

### Phase 2: Questionnaire surveys

#### Participants

The quantitative analyses were based on two cross‐sectional pen‐and‐paper questionnaire surveys within six hospitals in two regions in Sweden during November 2019 – mid January 2020, one directed to all CU managers (n = 276) and one to all nurses (n = 3,473). The response rates were 54% in both populations.

##### CU managers

Individual data for CU managers, in combination with data from the nurses aggregated to the CU level, were available for 139 cases (care units). Eighty‐nine percent of the participating managers were female, with a median age of 50 years (*m* = 50.9, *sd* = 7.4). Most respondents (85%) had been employed by their present regional healthcare organization for at least 10 years. Forty‐three percent had held the CU manager position for less than five years. The number of direct subordinates per CU manager ranged from 4–82, with a median of 35 (*m* = 35.1, *sd* = 17.6).

##### Nurses

Baseline questionnaire data were available for 1817 nurses (86% females). These nurses worked in 228 different care units, each containing between 1 and 47 respondents. The nurses' median age was 42 years (*m* = 42.4, *sd* = 12.2). About half (54%) had been employed by their present regional healthcare organization for more than 10 years. On average, they had been working as nurses for 15 years (*m* = 15.1, *sd* = 11.4). Sixty‐two percent had been working as nurses for at least 10 years.

#### Measures

##### CU managers' perceived job demands and resources

The CU managers' perceived job demands and resources were measured using items from the Gothenburg Manager Stress Inventory (GMSI) (Eklöf et al., 2010). Three different types of job demands were included: *organizational stressors* (three items, α = .73; sample item “insufficient resources to manage peak CU loads”), *role stressors* (three items, α = .72; sample item “discord between administrative work, organizational development tasks, and subordinate contacts”), and *subordinate stressors* (six items, α = .81; sample item “conflicts between subordinates”). Two types of resources were included: *superior managerial support* (four items, α = .85; sample item “my superior manager shows genuine interest in what I do and the problems I face as a manager”), and *peer support* (two items, *r* = .78; sample item “possibilities to discuss work with colleagues”). All demand and resource items had five fixed response alternatives, either ranging from “never/almost never” (1) to “always/almost always” (5), or ranging from “corresponds very poorly” (1) to “corresponds very well” (5).

##### CU managers' perceived organizational support

The CU managers' POS (8 items, α = .90) was measured using the short version of the Survey of perceived organizational support (Eisenberger et al., 1986; Neves and Eisenberger, 2012). Sample items included “the organization really cares about my well‐being”, and “the organization takes pride in my accomplishments at work”. There were six fixed response alternatives, ranging from “completely disagree” (1) to “completely agree” (6).

##### CU nurses' organizational climate of perceived organizational support

The CU nurses' climate of Perceived Organizational Support (POS‐climate) was measured based on the same eight items from the survey of perceived organizational support (Eisenberger et al., 1986; Neves and Eisenberger, 2012) as included in the managers' questionnaire, here reformulated to measure perceived shared climate (sample items: “the organization really cares about the employees' well‐being” and “the organization takes pride in its employees' accomplishments at work”) (α = .94). The intraclass correlation coefficient (ICC) was .27, indicating that about 27% of the variation in nurses' perceived POS‐climate could be attributed to climate perceptions shared within the CUs.

#### The measurement model

The measurement model consisted of the latent variables organizational stressors (three manifest indicators), role stressors (three manifest indicators), subordinate stressors (three manifest indicators), superior managerial support (four manifest indicators), peer support (two manifest indicators), managers' POS (three manifest indicators), and care unit nurses' POS climate (three indicators). For the variables subordinate stressors and managers' POS, unidimensional parceling (Kishton and Widaman, 1994) with random allocation into parcels was used in order to construct three indicators per latent variable. For the variable care unit POS climate, the nurses (*n* = 1817) within each CU (*n* = 139) were randomly allocated into one of three parcels. Their individual reports of the POS‐climate were then aggregated over each CU, resulting in three (aggregated) ratings of the POS climate for each CU. High values for these latent variables indicate high demands, high stressors, high CU managers' POS, and high CU nurses' POS climate, respectively.

#### Statistical analysis

**Fig. 2:**
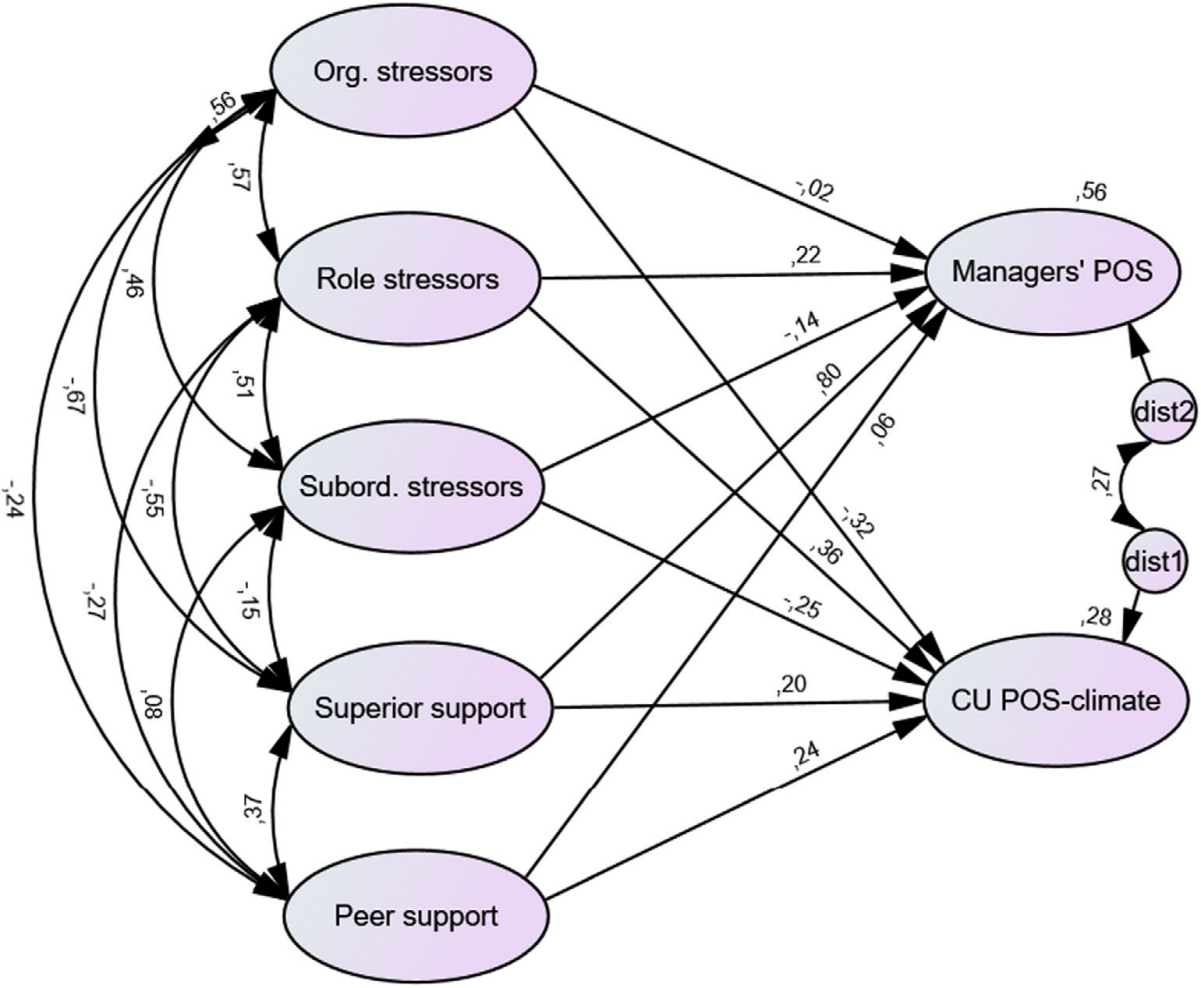
Conceptual model tested in the quantitative questionnaire surveys, with the resulting standardized estimates. Manifest indicators have been omitted from the figure. Note: Org. stressors: Organizational stressors; Subord. Stressors: Subordinate stressors; Superior support: Superior managerial support; CU POS‐climate: POS‐climate reported by the nurses at the care unit. [Colour figure can be viewed at wileyonlinelibrary.com]

The conceptual model of the hypothesized relationships between CU managers' demands and resources, CU managers' POS, and the nurses' POS‐climate at the CU, is shown in Fig. 2. The model contains the intercorrelated latent exogenous variables organizational stressors, role stressors, subordinate stressors, superior support, and peer support, all of which are hypothesized to directly influence the latent endogenous variables CU managers' POS and CU nurses' POS climate. The disturbance terms for managers' POS and nurses' POS climate were hypothesized to be correlated.

AMOS version 26 was used for model testing, employing the (full information) Maximum Likelihood (ML) estimator. Input data consisted of the raw data that were stored in SPSS. In order to set the scale for the latent variable, one factor loading per latent variable was set to 1.0. Model fit was evaluated using a combination of fit indices: the *χ* ^2^ statistic, which assesses the discrepancy between the sample covariance matrix and the model implied covariance matrix, with a statistically significant value relative to the degrees of freedom (*df*) indicating poor model fit; the RMSEA, with values of about .06 indicating close fit of the model to the data; and the CFI, with values greater than .95 indicating acceptable model fit (see, e.g., Hu and Bentler, 1999).

## RESULTS

### Interviews

#### Acting and organizing the work: Interactions with and within the care unit

All of the CU managers emphasized the need to be available for the nurses: “availability for the staff does not lead to dependence, it creates independence”. They stated that it enables provision of information, and participative development of the organization of work, opportunities to acknowledge and support individual team members, boost engagement, and facilitate the development of trust within the work team. Presence also enabled proactive identification of upcoming individual and collective needs, to “see what may be coming up, be one step ahead”. Availability could be achieved by participating in the daily care work at the unit, having the office situated at the CU, always having an “open door” for the staff, and participating at breaks and meetings. Most Swedish CU managers have a background in the nursing profession. Several of the informants described how they try to maintain their professional activity by regularly participating in the daily care work as nurses. This enhanced their credibility among the nursing staff and ensured deep knowledge about the nurses' needs and problems. Short, well‐structured, regular and frequent, participatory staff meetings were also mentioned as a means of supporting the nurses. At such meetings, all CU personnel present could be briefed about the current situation at the CU, and staffing could be flexibly restructured to adjust to immediate needs. Also, the yearly personal development dialogues with each employee helped the CU managers to ensure a minimum of availability and individual support for each employee.

An essential way to act supportively for the nurses was to contribute to their professional development by providing supervision and mentorship of recently graduated nurses, and supplementary education and training. This increased the total CU competence, provided opportunities for personal and career development for the nurses, and created a sense of security in an enlarged professional role. This contributed to a readiness to help each other across organizational boundaries, extending the possibility to allocate the nurses in a more flexible manner, adjusting staffing across similar teams or CUs according to immediate needs. Such flexible allocation of staff could also improve the continuity of care for patients. Flexible use of competence, in a way that ensured professional knowledge and security among the nurses, was further facilitated by good collegial cooperation between CU managers.

While the demand among the nurses for professional development was considered high, many CU managers experienced limited possibilities for the nurses to apply their extended competence through extended work content within the CU.

Competence exchange between healthcare professional groups was considered useful. This could relieve the nurses, but also physicians, of certain tasks that, under supervision, could equally well be performed by auxiliary nurses, and nurses, respectively, and thus provide professional development in the latter groups.

Virtually all of the CU managers stated that they were unable to be sufficiently available to the nurses. The reasons were heavy administrative workload, much time spent in meetings outside the unit, a large span of control, or an office situated far away from the CU. The lack of availability contributed to a constant sense of insufficiency and resulting stress that decreased availability even further; “no one wants to disturb a stressed manager”. The CU managers compensated for this by also being available to their staff outside working hours, which interfered with their own need for recuperation.

Several CU managers, but particularly those with many subordinates, compensated for a limited ability to be physically present and available for their staff, by having staff members as “tentacles” within the CU. These were often work environment representatives who had a role to protect the working environment at the CU. These staff members captured and forwarded information to the CU manager on events and individual needs that called for intervention from the CU manager.

The informants emphasized the importance of a trustful work unit culture with rich and open communication, high work engagement, and CU staff members providing each other with professional and social support. The CU managers acknowledged their role in contributing to such a culture. They also stated the importance of CU manager and members mutually supporting each other.

#### Preconditions from above: Managerial trust, communication, participation, and autonomy

The CU manager's influence over the working conditions at the CU was restrained by standardized policies, inducing bureaucratic control over everyday practice and a lack of responsiveness to the specific needs and context of the CU. This thwarted the CU managers' ability to ensure a sound workload for the nurses, and safeguard beneficial group dynamics.

Superior management's decisions and actions were considered decisive for the CU managers' ability to support nurses' POS. Standardized decisions and budgetary constraints disallowed considering contextual factors in the implementation. Although such decisions had a vast impact on the CUs, the CU managers were seldom consulted beforehand: “never during my many years as a CU manager has anyone asked me what I need to be able to do my job”.

The quality of the interactions and relations with the CU managers' immediate superior managers was fundamental for what could be done within directive limits. A constructive relation was characterized by mutual trust between the parties. This allowed the CU manager a substantial degree of autonomy in day‐to‐day work, and to exercise discretion in decisions regarding CU development and staffing. CU managers who had a trustful relationship with their immediate superior manager expressed that open discussions often helped them find new and constructive ways of thinking and acting.

The lack of coordination between different superior managerial functions was a problem. Concurrent and uncoordinated directives demanded CU manager actions with close deadlines for delivery. This created a lot of pressure and disrupted the CU managers' time plans, often at the cost of availability and staff‐oriented activities.

#### Preconditions from the side: Expertise, procedural guidance, and contextualized solutions from the administrative departments

The CU managers' interactions with and support from the administrative healthcare departments largely influenced their ability to support the nurses. High‐quality relations with staff at the Human Resource (HR) department were particularly important. The CU managers in general, and particularly those new in their role, had little knowledge about HR procedures and the laws that regulate them, and little experience of the digital administrative systems that were used. Therefore, the HR‐related work at the CU was stressful and required the CU managers to allocate a lot of time to learn and perform administrative tasks. This decreased their time for tasks directly aimed at supporting the nurses, and safeguarding a sound working environment. Some informants related that the HR staff had provided much‐needed support through legal expertise, guiding the manager through administrative procedures or sometimes performing such procedures. Such support released a lot of time for the CU managers to what they perceived as central tasks in their managerial role, rather than “getting stuck behind the computer”. HR support in recruitment processes had facilitated the manager's decision regarding whom to hire to safeguard effective group dynamics, and complement competencies at the unit, with great and long‐term influence on the CU work.

The CU managers' perceptions of the contribution from the HR department was largely reliant on the relation to and the mindset of the individual HR expert with whom they interacted. Long‐term relations with a specific HR expert created a comprehensive understanding from the HR staff member of the context‐specific conditions of the work at the specific CU, and the needs and abilities of each CU manager. However, high staff turn‐over at the HR department counteracted this. Also, increased specialization at the HR department, where each HR expert handled a narrow domain of issues, but across the entire hospital organization comprising several hospitals, implied that the CU managers had different HR contact persons depending on the type of errand. This decreased HR expert availability, their in‐depth knowledge of the specific CUs, and their possibility to develop profound and effective interpersonal relations with the CU managers: “there is more and more HR staff, but they provide less and less support”.

Several CU managers expressed an impression that the HR staff perceived their role as controlling rather than supporting the CU managers' work. The HR department was then perceived as acting solely on behalf of the top management, strictly enforcing the organization's policies, and unwilling or unable to consider context‐specific conditions at the CU.

#### Preconditions from within: Collegial dialogue and sharing of experience

Peer CU managers were an important source of support. One form of peer support was through shared leadership, where two CU managers equally shared the responsibility for the managerial tasks at one CU. Another solution was having an assistant manager to whom the responsibility for tasks, usually administrative and related to scheduling, was allocated, but with whom the CU manager could also openly discuss sensitive management matters. Both solutions freed up time to be present in the daily operations of the CU. Another important form of collegial support was frequently recurring meetings with all CU managers at the clinic, discussing common problems and openly sharing experience.

### Questionnaires

The tested model with resulting standardized estimates is depicted in Fig. 2. The model fit was acceptable (*χ* ^2^ = 258.2, *df* = 168, *p* < .001, CFI = 0.935, RMSEA = 0.062, 90% CI 0.047–0.077). Selected parameter estimates for the latent regression model are presented in Table 2.

**TABLE 2:** Selected parameters for the estimated model in the questionnaire study

			Estimate	S.E.	P	Standardized estimate
*Covariations*						
Superior_support	<−‐>	Role_stressors	−0.363	0.088	<0.001	−0.550
Superior_support	<−‐>	Organizational_stressors	−0.356	0.082	<0.001	−0.670
Superior_support	<−‐>	Peer_support	0.235	0.071	<0.001	0.371
Subordinate_stressors	<−‐>	Superior_support	−0.064	0.043	0.141	−0.152
Role_stressors	<−‐>	Organizational_stressors	0.250	0.069	<0.001	0.566
Role_stressors	<−‐>	Peer_support	−0.144	0.060	0.016	−0.274
Subordinate_stressors	<−‐>	Role_stressors	0.179	0.047	<0.001	0.512
Organizational_stressors	<−‐>	Peer_support	−0.103	0.048	0.032	−0.243
Subordinate_stressors	<−‐>	Organizational_stressors	0.130	0.038	<0.001	0.463
Subordinate_stressors	<−‐>	Peer_support	0.026	0.033	0.432	0.078
dist1(Care_unit_POSclimate)	<−‐>	dist2(Managers_POS)	0.062	0.030	0.042	0.269
*Direct effects*						
Care_unit_POSclimate	<−‐‐	Role_stressors	0.262	0.133	0.049	0.357
Care_unit_POSclimate	<−‐‐	Peer_support	0.183	0.085	0.031	0.241
Care_unit_POSclimate	<−‐‐	Organizational_stressors	−0.286	0.185	0.121	−0.315
Care_unit_POSclimate	<−‐‐	Superior_support	0.120	0.112	0.282	0.198
Care_unit_POSclimate	<−‐‐	Subordinate_stressors	−0.287	0.182	0.115	−0.250
Managers_POS	<−‐‐	Role_stressors	0.226	0.137	0.099	0.222
Managers_POS	<−‐‐	Peer_support	0.063	0.087	0.468	0.060
Managers_POS	<−‐‐	Organizational_stressors	−0.020	0.188	0.913	−0.016
Managers_POS	<−‐‐	Superior_support	0.671	0.131	<0.001	0.796
Managers_POS	<−‐‐	Subordinate_stressors	−0.224	0.189	0.236	−0.141

CU managers' demands, operationalized as organizational stressors, role stressors, and subordinate stressors, were all positively correlated. This means that CU managers who perceived a high amount of demands related to one of these sources generally also perceived a high amount of demands related to the other sources. CU managers' resources, operationalized as superior managerial support, and peer support, were positively correlated.

Organizational stressors as well as role stressors were negatively correlated with CU managers' resources; managers who perceived low access to support from their superior manager and peers also perceived high demands from the organization and the role as manager. However, subordinate stressors were not correlated with any of the resource variables.

Approximately 56% of the variation in managers' POS was explained by the exogenous variables included in the model (organizational stressors, role stressors, subordinate stressors, superior managerial support, and peer support). One strong predictor, superior managerial support, accounted for much of this variation (*β* = .80, *p* < .001), while none of the other predictors reached the significance level. However, role stressors showed a tendency to a positive relationship to managers' POS (*p* = .10). Thus, the managers' POS was predicted mainly by the support given by their superior manager.

About 28% of the variation in CU nurses' aggregated POS‐climate was explained by the CU managers' working conditions. Two predictors showed a significant effect. Peer manager support (*β* = .24, *p* = .03), as well as role stressors (*β* = .36, *p* = .05), showed a positive relationship with CU nurses' POS climate. Thus, in cases where the CU manager experienced support from peer managers, and where the CU manager perceived extensive role stress, their subordinate nurses reported more POS.

The disturbance term (residual) of CU managers' POS, and CU nurses' POS climate, were correlated (*r* = 0.27, *p* = .04). The correlation between CU managers' POS, and CU nurses' POS climate, estimated without predictors in the model, was *r* = 0.45, *p* < 0.001. This means that managers' and nurses' POS perceptions shared variation, and that some (but not all) of this shared variation was explained by the CU managers' working conditions (as operationalized in this study).

## DISCUSSION

### Methods and limitations

The present study applied a mixed‐methods design. In a critical review of mixed‐methods research in nursing, Bressan et al. (2017) stated that many studies applying such designs have serious flaws. Here, the mixed‐methods design was well motivated, since we approached complex interactions between different employees and managers at different organizational levels. Some of these issues were approached deductively, quantitatively testing relations between phenomena in a larger but sector‐specific sample. Other issues have previously received only scant investigation and called for an inductive, exploratory as well as interpretive and highly contextualized approach. The questionnaire study to CU managers could be subjected to common method bias, but the use of different methods and data sources limited this problem.

The quantitative part of the present study was cross‐sectional, so causality cannot be inferred. Causal interpretations must be investigated further using longitudinal data.

The study was restricted to two regional Swedish healthcare organizations, which may limit the transferability of the results. However, in both the quantitative and the qualitative parts, we strived for maximum variation, with participation of CUs within a wide range of medical specialties in different regions and hospitals. The quantitative questionnaire study was directed to all nurses employed at these hospitals. The response rate at the CU level was acceptable and all types of specialized healthcare in the target population were represented. We also sought maximum variation among the individual participants in the qualitative study concerning medical specialty care, age, and seniority.

The demands and resources addressed in the quantitative study were limited and it is probable that also other types of demands and resources will influence CU managers' POS and their ability to work effectively as leaders. In the selection of demands and resources, we leaned on previous research on working conditions for leaders in Swedish healthcare (Eklöf et al., 2010). The qualitative results specifying the type and sources of support the CU managers needed further validated the choice of quantitative measures.

### General discussion

In the qualitative interviews, the CU managers described how they act and try to organize their work to promote the development of nurses' POS. They stressed the importance of being available to their staff, how they tried to achieve this and compensated for insufficient availability.

The quantitative part of the study showed that the CU managers' working conditions were related directly to the CU POS climate, as reported by the nurses. One important working condition here was the CU managers' support from their peers. This corroborates the finding in the qualitative part of this study. Interestingly, the CU managers support from their superior manager was important for the CU managers' own POS, but did not contribute directly to the CU nurses' POS climate.

We observed a positive relationship between the CU managers' role stress and the CU POS climate reported by the nurses. We believe that this somewhat unexpected finding indicates that CU managers who make substantial efforts to be available to and directly support their staff are likely to experience more role conflicts than those who focus more on administrative tasks and standardized organizational development. Being highly available to the staff and actively provide daily support may come at the cost of role conflicts for the CU manager. Spending time and effort to meet individual and collective needs may lead to role overload. It is important to follow up on such possible interacting effects in future research.

The working conditions for the CU managers were decisive for their own POS, as well as for their ability to support the development of nurses' POS. The qualitative part of the study emphasized the CU managers need for responsive support from leaders and administrative staff, allowing the CU managers to implement new directives in a manner that was adapted to the specific needs and conditions at the respective CU. The quantitative part of the study indicated the importance of support from their superior manager for the CU managers' POS. These results are concordant with those of a comprehensive meta‐analysis (Kurtessis et al., 2017), where resources in terms of autonomy and work influence were identified as important antecedents of POS. In addition, the qualitative results of our study showed that CU managers' autonomy and possibility to influence and adapt decisions to the specific needs at the CU were also important for the ability of CU managers' to provide the nurses with organizational support. Further, the results from the quantitative study indicated that the CU managers' POS was directly associated with the nurses' POS climate, aggregated to the CU level. In some sense, such a positive association may be expected given the operationalization of the POS‐climate. Both CU managers and nurses were instructed to rate perceptions of “the organization”, which included both the immediate supervisor and more superior managerial levels. Thus, to some extent the CU managers and CU nurses assessed the same underlying construct. However, previous research based on qualitative interviews with the nurses indicated that the nurses' knowledge and perceptions of the superior healthcare management was diffuse (Gadolin et al., 2021). This suggests that the nurses' ratings of the POS climate in the present study were primarily based on their perceptions of the CU manager as the organizational representative. Therefore, we wish to highlight the importance of CU managers' POS for the nurses' POS‐climate. The results are concordant with those of others (Shanock and Eisenberger, 2006), who found that supervisors' individual POS was related to their subordinates individual POS, and that this relation was mediated through the subordinates perceived supervisor support.

There is convincing evidence in previous literature for the positive effect of POS on employee health and well‐being, their intentions to stay in their job, as well as work performance (Kurtessis et al., 2017; Shanock and Eisenberger, 2006). Our results indicate that the demands on and resources for CU managers in healthcare influence not only their own health and well‐being, but also their ability to support the nurses' POS. Consequently, the quality of CU managers' working conditions is decisive for the quality and efficiency of the healthcare that is delivered “on the floor”. Demands in terms of work overload, lack of autonomy and influence, and unclear or conflicting demands were negatively related to CU managers' POS, while demands stemming from the subordinates did not significantly influence CU managers' POS. The results of the qualitative interviews gave rich, contextualized, and concrete descriptions on how CU managers act and (try to) organize their work, to provide the nurses with organizational support. These results also chiseled out the type of support the CU managers need to enable this work, and the major sources of such support.

Regarding support from the administrative departments, the informants particularly emphasized the importance of high‐quality relations with staff at the HR department. The CU managers in general, and particularly those new in their role, expressed little knowledge about HR procedures and the laws that regulate them. In general, they also had little previous knowledge of the digital administrative systems that were used by the management. This points to a need for more and better education in HR legislation, procedures, and tools to CU managers before they enter their new role, and to also provide such complementary education to the more senior CU managers. Better formal knowledge on these issues could relieve a lot of stress on the CU managers, as well as enable them to better spend time on their direct CU leadership.

Support from the HR experts for strategic recruitments to the CUs was held forth as imperative, but this could be hampered by the organization of the HR departments. The informants stated that the HR experts were increasingly specialized in narrow HR domains, serving a large number of CUs with such support. This specialization came at the price of lost contextual knowledge on the preconditions and needs at the particular CUs, and on the competence of the respective CU managers. The CU managers expressed that long‐term relations with a specific HR expert were effective and efficient, but several of them stated that there had been extensive staff turnover at the HR department. If this is the case, the reasons for such turnover need to be investigated. Is work at a hospital HR department merely viewed as a stepping stone in an HR career? How does the HR staff perceive their role and the conditions that decide their ability to perform their job meaningfully?

The CU managers perceived little discretion in decision making when aiming to adapt overarching organizational policies to the operations of the CU. They needed support from the administrative departments that was well adjusted to their specific needs. Such adaptions were hampered by standardized directives. A common view among the CU managers was that the role of the HR staff was to control, rather than support, their work. The HR department was then perceived as acting solely on behalf of the top healthcare management, strictly enforcing the organization's policies, and unwilling or unable to take context‐specific conditions at the CU into consideration. One could speculate that this indicates a profound problem related to the governance of healthcare organizations, where the budgetary limits of the organization are set by imperturbable political decisions that do not correspond to the actual healthcare needs. In such an organization, the management may strive to avoid the confrontation with the actual needs and the resulting conflicts between budgetary constraints and ensuring high quality of care, by delegating the responsibility to implement the decisions (Tengelin et al., 2011). The economy and HR departments receive the responsibility to implement decisions at the CUs, refer to management decisions, and do not have the authority to be flexible. This enforcing position may also counteract close relations between the HR experts and the CU managers. It may even help to explain a high turnover among the healthcare HR staff.

## CONCLUSION

The present study has highlighted that the CU managers considered that being present at the CU and being highly available to their staff were central for their ability to provide the nurses with organizational support. The CU managers held forth their own support from three organizational levels as decisive for their ability to act and organize their work in a facilitating manner. Firstly, support from their superior management was provided when the relations were based on mutual trust and open communication, where the CU manager was invited to participate in decision making processes and allowed a certain degree of autonomy in decisions concerning the operations of their own CU. Secondly, the administrative departments offered support when providing expertise, procedural guidance, and contextualized solutions anchored in well‐developed inter‐personal relationships with the CU manager. Thirdly, support from the peer CU managers was provided through a frequent collegial dialog and sharing of experience, and through co‐management of the CU. The study further showed that support from the superior management was related to the CU managers' own POS, and that this, in turn, was associated with the nurses' shared organizational POS climate at the unit. The results emphasize the centrality of providing enabling working conditions for the CU managers is order to support the development of the nurses' Perceived Organizational Support.

## Funding statement

This research was funded by AFA Försäkring grant no. 180085.

## Conflict of interest

None.

## Ethics approval statement

Ethics approval was obtained from the Regional Ethics Committee in Gothenburg (no. 264–18), and the study was performed in accordance with the ethical standards laid down in the 1964 Declaration of Helsinki and its later amendments. Informed consent was provided by all participants prior to inclusion in the study.

## Patient consent statement

N/A

## Permission to reproduce material from other sources

N/A

## Clinical trial registration

N/A

## Data Availability

The data that support the findings of this study are available from the corresponding author upon reasonable request.
